# Methyl­phospho­nic acid, CH_3_PO(OH)_2_

**DOI:** 10.1107/S1600536814003572

**Published:** 2014-02-26

**Authors:** Hans Reuter, Martin Reichelt

**Affiliations:** a Institute of Chemistry of New Materials, University of Osnabrück, Barbarastrasse 7, 49069 Osnabrück, Germany

## Abstract

The asymmetric unit of the title compound, CH_5_O_3_P, contains two independent mol­ecules with nearly identical bond lengths and angles. In the crystal, each of the mol­ecules acts as acceptor (P=O) and donor (P—OH) of four hydrogen bonds to three adjacent mol­ecules, resulting in the formation of two different bilayers (one for each mol­ecule) stacked perpendicular to the *a* axis in the crystal.

## Related literature   

For organic and inorganic tin compounds of methyl phospho­nic acid, see: Adair *et al.* (1998 ▶); Ribot *et al.* (2001 ▶). For structural data on phenyl phospho­nic acid, see: Weakley (1976 ▶); Mahmoudkhani & Langer (2002 ▶). For a brief communication on the unit-cell parameters of methyl phospho­nic acid, see: Kodolov *et al.* (1977 ▶). For comparative studies of dimeric carb­oxy­lic acids, see: Allan *et al.* (2000) ▶; Bruno & Randaccio (1980 ▶).
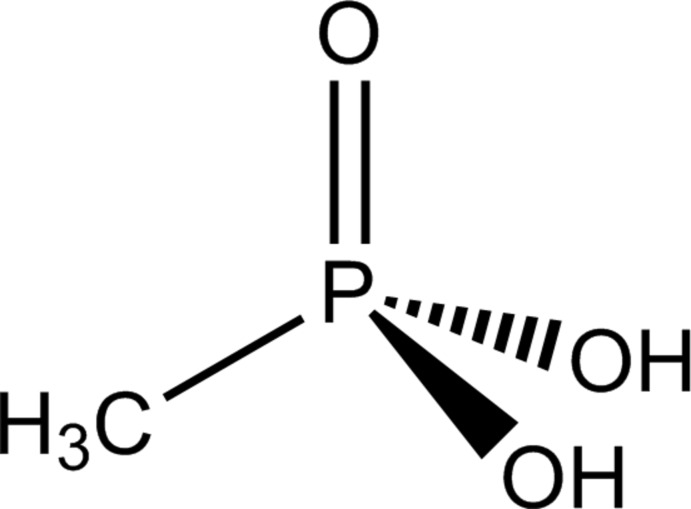


## Experimental   

### 

#### Crystal data  

CH_5_O_3_P
*M* *_r_* = 96.02
Monoclinic, 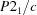

*a* = 15.1015 (8) Å
*b* = 5.7704 (3) Å
*c* = 9.9549 (6) Å
β = 108.262 (2)°
*V* = 823.79 (8) Å^3^
*Z* = 8
Mo *K*α radiation
μ = 0.51 mm^−1^
*T* = 200 K
0.45 × 0.26 × 0.12 mm

#### Data collection  

Bruker APEXII CCD diffractometer
Absorption correction: multi-scan (*SADABS*; Bruker, 2009 ▶) *T*_min_ = 0.805, *T*_max_ = 0.942
58588 measured reflections
1989 independent reflections
1763 reflections with *I* > 2σ(*I*)
*R*_int_ = 0.075

#### Refinement  

*R*[*F* ^2^ > 2σ(*F* ^2^)] = 0.028
*wR*(*F* ^2^) = 0.087
*S* = 1.08
1989 reflections
97 parameters
H-atom parameters constrained
Δρ_max_ = 0.33 e Å^−3^
Δρ_min_ = −0.35 e Å^−3^

### 

Data collection: *APEX2* (Bruker, 2009 ▶); cell refinement: *SAINT* (Bruker, 2009 ▶); data reduction: *SAINT*; program(s) used to solve structure: *SHELXS97* (Sheldrick, 2008 ▶); program(s) used to refine structure: *SHELXL97* (Sheldrick, 2008 ▶); molecular graphics: *DIAMOND* (Brandenburg, 2006 ▶) and *Mercury* (Macrae *et al.*, 2008 ▶); software used to prepare material for publication: *SHELXTL* (Sheldrick, 2008 ▶).

## Supplementary Material

Crystal structure: contains datablock(s) I, New_Global_Publ_Block. DOI: 10.1107/S1600536814003572/hg5383sup1.cif

Structure factors: contains datablock(s) I. DOI: 10.1107/S1600536814003572/hg5383Isup2.hkl

Supporting information file. DOI: 10.1107/S1600536814003572/hg5383Isup3.cml

CCDC reference: 987419

Additional supporting information:  crystallographic information; 3D view; checkCIF report

## Figures and Tables

**Table 1:** Selected bond lengths (Å)

P1—O13	1.4993 (11)
P1—O11	1.5441 (11)
P1—O12	1.5443 (12)
P1—C1	1.7586 (17)
P2—O23	1.4989 (11)
P2—O21	1.5478 (11)
P2—O22	1.5504 (12)
P2—C2	1.7612 (17)

**Table 2:** Hydrogen-bond geometry (Å, °)

*D*—H⋯*A*	*D*—H	H⋯*A*	*D*⋯*A*	*D*—H⋯*A*
O22—H22⋯O23^i^	0.96	1.59	2.5528 (15)	180
O21—H21⋯O23^ii^	0.96	1.65	2.5768 (16)	161
O12—H12⋯O13^iii^	0.96	1.61	2.5649 (15)	174
O11—H11⋯O13^iv^	0.96	1.62	2.5671 (16)	169

Symmetry codes: (i) 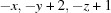; (ii) 
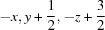
; (iii) 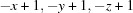; (iv) 
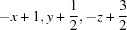
.

**Fig. 1.**
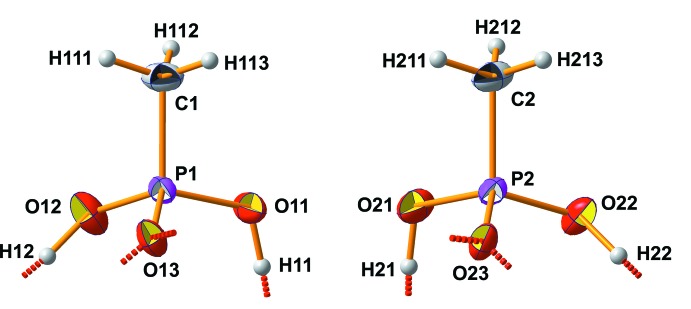
Ball-and-stick model of the two molecules in the asymmetric unit of the title compound with the atomic numbering scheme used; with exception of the hydrogen atoms, which are shown as spheres with a common isotropic radius, all other atoms are represented as thermal displacement ellipsoids at the 50% probability level; hydrogen bonds are indicated by broken sticks (red).

**Fig. 2.**
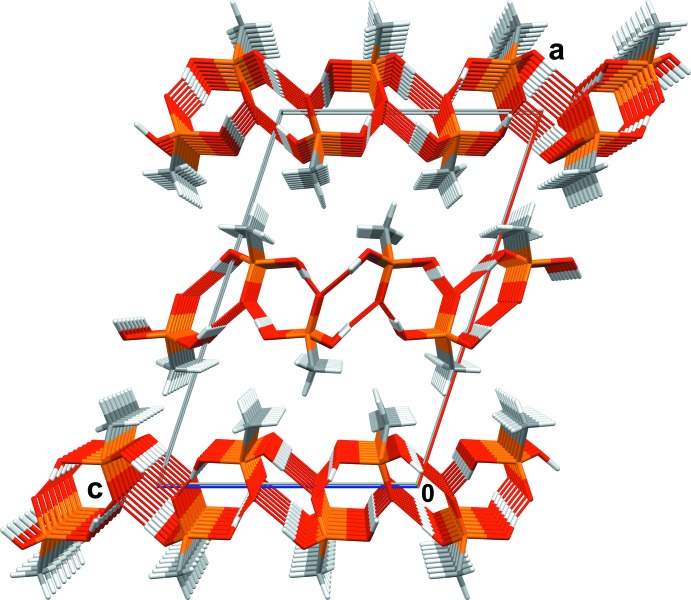
Simplified ball-and-stick model of the crystal packing of MePO(OH)2 showing its bilayer structure; color code: oxygen = red, phosphorous = orange, carbon = dark grey, hydrogen = light grey; hydrogen bonds are shown as broken sticks (red).

**Fig. 3.**
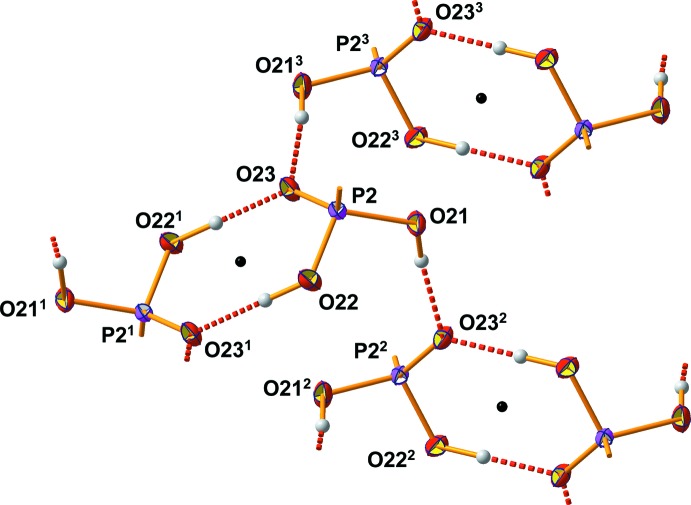
Hydrogen bonding system in the bilayer of MePO(OH)2 resulting from molecule 2; with exception of the hydrogen atoms, which are shown as spheres with a common isotropic radius, all other atoms are represented as thermal displacement ellipsoids at the 50% probability level; methyl groups are omitted for clarity; hydrogen bonds are indicated by broken sticks (red); small black balls = center of symmetry; symmetry transformations used to generate equivalent atoms: 1) -x, 2 - y, 1 - z; 2) -x, 1/2 + y, 3/2 - z; 3) -x, -1/2 + y, 3/2 - z.

## supplementary crystallographic information

### 1.1. Synthesis and crystallization

Single crystals of methyl phospho­nic acid (Merck-Schuchardt) were obtained as side products from several experiments when we tried to growth singles crystals of diorganotin(IV) methyl phospho­nates by solvent evaporation. A suitable single crystal was selected under a polarization microscope and mounted on a 50 µm MicroMesh MiTeGen Micromount^TM^ using FROMBLIN Y perfluoro­polyether (LVAC 16/6, Aldrich).

### 1.2. Refinement

All hydrogen atoms could be localized in difference Fourier synthesis. Those of the methyl groups were idealized and refined at calculated positions riding on the carbon atoms with C—H distance of 0.98 Å. Those of the hydroxyl groups were refined with respect to a common O—H distance of 0.96 Å before they were fixed and allowed to ride on the corresponding oxygen atoms. For the hydrogen atoms of each methyl group a common isotropic displacement parameter was refined as well as one common isotropic displacement parameter for the hydrogen atoms of all hydroxyl groups.

### 2. Results and discussion

Methyl­phospho­nic acid, CH_3_PO(OH)_2_, is a well-established reagent in inorganic as well as in organometallic chemistry forming numerous salts respectively coordination compounds with a lot of different metals and organometallic fragments. For an example, from tin(II) the synthesis and structural characterization of the mixed methyl­phospho­nate oxalate Sn_2_(MePO_3_)(C_2_O_4_) was described by Adair *et al.* (1998) whereas diorganotin(IV) moieties will give rise to the formation of dimeric species like [Bu_2_Sn(MePO(OH))_2_]_2_ as was shown by Ribot *et al.* (2001). During a systematic study on the complex formation of methyl­phospho­nic acid towards other organotin(IV) compounds we became aware, that the crystal structure of the title compound was never determined in detail. Only Kodolov *et al.* (1977) referred in a brief communication to the cell parameters of a monoclinic unit cell but no atomic coordinates were given.

During our studies, we never were able to confirm these cell parameters, although we also found a monoclinic unit cell. Moreover, we observed a strong broadening and multiplication of the reflections on cooling down the crystal to 100 K. For this reason, we present the results of a measurement at T = 200 K, before reflection broadening started.

The asymmetric unit of the title compound consists of two crystallographic independent molecules (Fig. 1) with nearly identical geometrical parameters (Tab. 1). These values correspond very well with those of phenyl phospho­nic acid that was determined by Mahmoudkhani *et al.* (2002) at T = 183 (2) K [d(P—O) = 1.536 (2) Å, 1.555 (2) Å; d(P=O) = 1.506 (2) Å and d(P—C) = 1.782 (3) Å] and Weakley (1976) at ambient temperature [d(P—O) = 1.539 (3) Å, 1.550 (4) Å; d(P=O) = 1.496 (4) Å and d(P—C) = 1.773 (5) Å]. With respect to bond angles at phospho­rous, tetra­hedral environment is much more distorted in the present compound [103.46 (8)° -112.94 (7)°] than in the corresponding phenyl compound [106.9 (2)° - 112.1 (2)°, Weakley (1976); 107.5 (1)°-111.7 (1)°, Mahmoudkhani *et al.* (2002)]

Each of the two molecules in the asymmetric unit is involved into four hydrogen bonds with molecules of the same kind giving rise to two different bilayers stacked perpendicular to the crystallographic a-axis (Fig. 2). Within each bilayer, each molecule of the upper layer is hydrogen bonded to three molecules of the lower layer and vice versa. In summary, the arrangement of the phospho­rous atoms in each bilayer corresponds to the arrangement of the arsenic atoms in the double-layer structure of grey arsenic consisting of an extended network of fused, six-membered rings of arsenic atoms with chair conformation.

Two of the four hydrogen bonds of each molecule result from centrosymmetric dimers (Fig. 3) in which one hydroxyl group (O22—H22) acts as donor and the double-bonded oxygen atom (O22) as acceptor for almost linear hydrogen bonds with a bond angle H···O—P of about 120° at the double bonded oxygen atom. These dimers are very similar those found in carbonic acids like propionic acid (Allan *et al.* 2000) or benzoic acid (Bruno & Randaccio, 1980). The double bonded oxygen atoms, additionally, are involved into a second pair of inter­molecular hydrogen bonds to the second hydroxyl group. These hydrogen bonds, also being less linear as the former one, are of similar length (Tab. 2).

### Crystal data

**Table:**

CH_5_O_3_P	*F*(000) = 400
*M**_r_* = 96.02	*D*_x_ = 1.548 Mg m^−^^3^
Monoclinic, *P*2_1_/*c*	Mo *K*α radiation, λ = 0.71073 Å
Hall symbol: -P 2ybc	Cell parameters from 9876 reflections
*a* = 15.1015 (8) Å	θ = 2.8–28.7°
*b* = 5.7704 (3) Å	µ = 0.51 mm^−^^1^
*c* = 9.9549 (6) Å	*T* = 200 K
β = 108.262 (2)°	Rhomboidal plate, colourless
*V* = 823.79 (8) Å^3^	0.45 × 0.26 × 0.12 mm
*Z* = 8	

### Data collection

**Table:**

Bruker APEXII CCD diffractometer	1989 independent reflections
Radiation source: fine-focus sealed tube	1763 reflections with *I* > 2σ(*I*)
Graphite monochromator	*R*_int_ = 0.075
φ and ω scans	θ_max_ = 28.0°, θ_min_ = 2.8°
Absorption correction: multi-scan (*SADABS*; Bruker, 2009)	*h* = −19→19
*T*_min_ = 0.805, *T*_max_ = 0.942	*k* = −7→7
58588 measured reflections	*l* = −13→13

### Refinement

**Table:**

Refinement on *F*^2^	Secondary atom site location: difference Fourier map
Least-squares matrix: full	Hydrogen site location: inferred from neighbouring sites
*R*[*F*^2^ > 2σ(*F*^2^)] = 0.028	H-atom parameters constrained
*wR*(*F*^2^) = 0.087	*w* = 1/[σ^2^(*F*_o_^2^) + (0.0446*P*)^2^ + 0.3474*P*] where *P* = (*F*_o_^2^ + 2*F*_c_^2^)/3
*S* = 1.08	(Δ/σ)_max_ = 0.001
1989 reflections	Δρ_max_ = 0.33 e Å^−^^3^
97 parameters	Δρ_min_ = −0.35 e Å^−^^3^
0 restraints	Extinction correction: *SHELXL97* (Sheldrick, 2008), Fc^*^=kFc[1+0.001xFc^2^λ^3^/sin(2θ)]^-1/4^
Primary atom site location: structure-invariant direct methods	Extinction coefficient: 0.0089 (18)

### Special details

**Table:**

Geometry. All e.s.d.'s (except the e.s.d. in the dihedral angle between two l.s. planes) are estimated using the full covariance matrix. The cell e.s.d.'s are taken into account individually in the estimation of e.s.d.'s in distances, angles and torsion angles; correlations between e.s.d.'s in cell parameters are only used when they are defined by crystal symmetry. An approximate (isotropic) treatment of cell e.s.d.'s is used for estimating e.s.d.'s involving l.s. planes.
Refinement. Refinement of *F*^2^ against ALL reflections. The weighted *R*-factor *wR* and goodness of fit *S* are based on *F*^2^, conventional *R*-factors *R* are based on *F*, with *F* set to zero for negative *F*^2^. The threshold expression of *F*^2^ > σ(*F*^2^) is used only for calculating *R*-factors(gt) *etc*. and is not relevant to the choice of reflections for refinement. *R*-factors based on *F*^2^ are statistically about twice as large as those based on *F*, and *R*- factors based on ALL data will be even larger.

### Fractional atomic coordinates and isotropic or equivalent isotropic displacement parameters (Å^2^)

**Table:**

	*x*	*y*	*z*	*U*_iso_*/*U*_eq_	
P1	0.41034 (3)	0.35924 (6)	0.60908 (4)	0.02670 (13)	
O11	0.40297 (9)	0.4245 (2)	0.75562 (13)	0.0446 (3)	
H11	0.4433	0.5450	0.8066	0.070 (4)*	
O12	0.38152 (8)	0.5724 (2)	0.51127 (14)	0.0466 (3)	
H12	0.4251	0.6363	0.4691	0.070 (4)*	
O13	0.50590 (7)	0.2779 (2)	0.61564 (12)	0.0365 (3)	
C1	0.32469 (13)	0.1438 (3)	0.5486 (2)	0.0480 (5)	
H111	0.3212	0.0982	0.4522	0.083 (5)*	
H112	0.2641	0.2045	0.5488	0.083 (5)*	
H113	0.3411	0.0086	0.6111	0.083 (5)*	
P2	0.09029 (3)	0.86636 (6)	0.69483 (4)	0.02720 (13)	
O21	0.09504 (9)	0.9190 (2)	0.84939 (12)	0.0459 (3)	
H21	0.0495	1.0299	0.8568	0.070 (4)*	
O22	0.11315 (8)	1.0935 (2)	0.62934 (12)	0.0414 (3)	
H22	0.0712	1.1440	0.5404	0.070 (4)*	
O23	−0.00227 (7)	0.7710 (2)	0.60798 (11)	0.0377 (3)	
C2	0.18264 (13)	0.6703 (3)	0.71256 (18)	0.0436 (4)	
H211	0.1700	0.5260	0.7551	0.069 (4)*	
H212	0.2409	0.7385	0.7733	0.069 (4)*	
H213	0.1885	0.6373	0.6191	0.069 (4)*	

### Atomic displacement parameters (Å^2^)

**Table:**

	*U*^11^	*U*^22^	*U*^33^	*U*^12^	*U*^13^	*U*^23^
P1	0.0301 (2)	0.0254 (2)	0.0277 (2)	−0.00282 (13)	0.01342 (15)	−0.00150 (13)
O11	0.0563 (7)	0.0486 (7)	0.0382 (6)	−0.0219 (6)	0.0283 (6)	−0.0172 (5)
O12	0.0384 (6)	0.0457 (7)	0.0635 (8)	0.0146 (5)	0.0269 (6)	0.0244 (6)
O13	0.0345 (6)	0.0381 (6)	0.0405 (6)	0.0082 (5)	0.0172 (5)	0.0157 (5)
C1	0.0487 (10)	0.0457 (10)	0.0550 (11)	−0.0194 (8)	0.0239 (9)	−0.0222 (8)
P2	0.0305 (2)	0.0268 (2)	0.0223 (2)	0.00449 (13)	0.00533 (15)	0.00254 (12)
O21	0.0605 (8)	0.0509 (7)	0.0246 (5)	0.0224 (6)	0.0108 (5)	0.0001 (5)
O22	0.0369 (6)	0.0361 (6)	0.0413 (6)	−0.0076 (5)	−0.0019 (5)	0.0111 (5)
O23	0.0345 (6)	0.0411 (6)	0.0329 (5)	−0.0065 (5)	0.0038 (4)	0.0135 (5)
C2	0.0449 (9)	0.0420 (9)	0.0392 (9)	0.0169 (8)	0.0065 (7)	−0.0033 (7)

### Geometric parameters (Å, º)

**Table:**

P1—O13	1.4993 (11)	P2—O23	1.4989 (11)
P1—O11	1.5441 (11)	P2—O21	1.5478 (11)
P1—O12	1.5443 (12)	P2—O22	1.5504 (12)
P1—C1	1.7586 (17)	P2—C2	1.7612 (17)
O11—H11	0.9583	O21—H21	0.9584
O12—H12	0.9584	O22—H22	0.9580
C1—H111	0.9800	C2—H211	0.9800
C1—H112	0.9800	C2—H212	0.9800
C1—H113	0.9800	C2—H213	0.9800
			
O13—P1—O11	112.86 (7)	O23—P2—O21	112.94 (7)
O13—P1—O12	110.73 (6)	O23—P2—O22	110.99 (6)
O11—P1—O12	108.17 (8)	O21—P2—O22	107.75 (7)
O13—P1—C1	112.85 (8)	O23—P2—C2	112.83 (8)
O11—P1—C1	103.46 (8)	O21—P2—C2	103.76 (7)
O12—P1—C1	108.39 (9)	O22—P2—C2	108.15 (9)
P1—O11—H11	117.1	P2—O21—H21	113.4
P1—O12—H12	119.1	P2—O22—H22	118.4
P1—C1—H111	109.5	P2—C2—H211	109.5
P1—C1—H112	109.5	P2—C2—H212	109.5
H111—C1—H112	109.5	H211—C2—H212	109.5
P1—C1—H113	109.5	P2—C2—H213	109.5
H111—C1—H113	109.5	H211—C2—H213	109.5
H112—C1—H113	109.5	H212—C2—H213	109.5

### Hydrogen-bond geometry (Å, º)

**Table:**

*D*—H···*A*	*D*—H	H···*A*	*D*···*A*	*D*—H···*A*
O22—H22···O23^i^	0.96	1.59	2.5528 (15)	180
O21—H21···O23^ii^	0.96	1.65	2.5768 (16)	161
O12—H12···O13^iii^	0.96	1.61	2.5649 (15)	174
O11—H11···O13^iv^	0.96	1.62	2.5671 (16)	169

Symmetry codes: (i) −*x*, −*y*+2, −*z*+1; (ii) −*x*, *y*+1/2, −*z*+3/2; (iii) −*x*+1, −*y*+1, −*z*+1; (iv) −*x*+1, *y*+1/2, −*z*+3/2.
